# Editorial: Vaccines and Immunostimulants for Finfish

**DOI:** 10.3389/fimmu.2020.573771

**Published:** 2020-09-29

**Authors:** Hetron M. Munang'andu, Irene Salinas, Carolina Tafalla, Roy Ambli Dalmo

**Affiliations:** ^1^Faculty of Veterinary Medicine, Norwegian University of Life Sciences, Oslo, Norway; ^2^Biology Department, University of New Mexico, Albuquerque, NM, United States; ^3^Animal Health Research Center (Centro de Investigación en Sanidad Animal - Instituto Nacional De Investigaciones Agrarias), Madrid, Spain; ^4^Universitet i Tromsø – The Arctic University of Norway, Tromsø, Norway

**Keywords:** vaccines, immunostimulants, antigens, correlates of protection, immune training

The expansion of intensive fish farming in recent decades has led to increase in the number of fish diseases (1, 2). This is partly because high stocking densities used in intensive farming cause stress making fish more susceptible to diseases and lead to high disease transmission index due increased contact among fish. These factors call for the need of protective vaccines (3). Understanding how the fish immune system responds to vaccines and immunostimulants/adjuvants is crucial for the rational development of efficient vaccines. These studies should guide the design and selection of effective immunostimulants and vaccine antigens that are efficiently taken up, processed and presented to cells of the adaptive immune system. Another priority of the field is the identification of optimal correlates of protective immunity that may be used as benchmarks in the design of long-term protective fish vaccines. This has traditionally been a challenge due to poor correlation between protection and antibody responses (4), but new emerging tools should complement traditional serological assays. The compilation of studies presented in this Special Issue provides an updated look on various immunostimulants and vaccine antigens relevant to farmed fish health. In addition, these papers also provide an update on recent discoveries in fish immunology that could aid in the design of highly protective vaccines for finfish, as well as to establish correlates of protection for these vaccines. Thus, we envisage that this Special Issue provides novel valuable insights essential for all researches working on the development and implementation of immune strategies that reduce the impact of infectious diseases in aquaculture.

## Immunostimulants and Innate Immune Responses

Immunostimulants (IMM) have historically been defined as substances that have the capacity to increase innate immune responses (5). But because innate responses have been shown to greatly influence adaptive responses, their administration along with vaccine antigens also influences the adaptive immune responses specifically mounted to the antigen. In this case, we refer to these immunostimulants as adjuvants. The term adjuvant is from the latin “adjuvare” meaning to help. As an example, immunostimulants such as certain oligosaccharides, CpGs, LPS, β-glucans, or flagellin can be administered parenterally or orally to fish without a vaccine to increase the general immune status of the fish (6) or they can be given together with a vaccine antigen (7). To date, commercial vaccines mostly use mineral oils as adjuvants (5). These mineral oils help induce a more robust immune response than the antigen alone by increasing the immunogenicity of weak antigens, prolonging the duration of antigen release at the injection site, and also stimulating and modulating adaptive immune responses. The use of IMM as adjuvants has been exclusively assayed experimentally, but they need to be more focused on specific immune functions that are beneficial for a defined antigen, without provoking a massive pro-inflammatory response that can lead to unwanted side effects. Thus, IMM can for example promote expression and secretion of cytokines, soluble factors and enzymes that modulate migration of leukocytes to antigen deposition sites, enhance antigen uptake and the intracellular processing and presentation of antigen to cells of the adaptive immune systems (5). Among a broad range of IMM, chitosan oligosaccharides (COSs), synthetic pituitary adenylate cyclase-activating polypeptide (PACAP), CpGs, and flagellin have all been shown to stimulate the effector functions of monocytes, macrophages, dendritic-like, and B cells in fish. The study performed by Wu et al. showed that activation of macrophages using three different molecular weight COSs (~500, ~1,000, and 2,000~3,000 Da) correlated with different chitosan oligosaccharides molecular weights (COS-MWs) of which the COS-MW with highest molecular weight had the highest activation of macrophages. In another study, Semple et al. showed that monocyte/macrophage- RTS11 cells pre-treated with PACAP 24 h before infection, severely inhibited *Flavobacterium psychrophilum* growth and increased expression of pro-inflammatory cytokines together with PACAP receptors in a dose-dependent manner. Simón et al. showed that CpGs increased the survival of IgM^+^ B cells, induced their proliferation and differentiation to plasmablasts/plasma cells, upregulated MHC-II and co-stimulatory molecules, and increased their phagocytic capacity while Iliev et al. showed that CpG stimulation transformed the rainbow trout primary phagocytes into dendritic-like-cells exhibiting long branching pseudopodia. These dendritic-like cells expressed high pro-inflammatory genes belonging to a Th17 response (IL-17A/F1) and a Th2 response (IL-4/13) where IL-10 and IFNγ was downregulated. Both studies demonstrated that CpGs are capable of modulating both the innate and adaptive branches of the immune system in fish, thus pointing to these molecules as optimal vaccine adjuvants. To gain further insight on how these CpGs are recognized by the host, Lai et al. performed an in depth study that points out that recognition of CpGs in fish is carried out by two sensors, namely Toll-like receptor 9 (TLR9) and TLR21, consequently demonstrating that the signaling of CpGs is more complex in fish than in mammals that only use TLR9 as a CpG sensor. Wangkahart et al. showed that *Yersinia ruckeri* flagellin upregulated several pro-inflammatory cytokines, acute phase proteins, antimicrobial peptides and complement genes in multiple tissues after *in vivo* administration, results that point to flagellin as another potent immunostimulant/adjuvant for fish aquaculture.

Developing *in vitro* models for studying immune responses to vaccine antigens or to IMM, is of great interest, to reduce the use of animals and pre-evaluate the potential of these substances through an easy method. In this sense, Wang J. et al. developed an *in vitro* model for studying responses to immunostimulation in the gut using rainbow trout intestinal epithelial cells (RTgutGC) propagated on conventional culture plates and transwell membranes, which they used to evaluate gut responses to stimulation by LPS and three functional feed ingredients namely; (i) mannanoligosaccharides (MOS), (ii) β-glucans, and (iii) nucleotides. MOS was found to be the most potent immunostimulant that upregulated several inflammatory cytokines such IL-1β, IL-6, IL-8, TNFα, and TGFβ at similar levels with LPS while nucleotides and β-glucans only upregulated IL-1β and IL-8. Overall, RTgutGC cells had features characteristic of functional intestinal cells and therefore offer a useful *in vitro* model useful for evaluating the effects of IMM and feed ingredients on gut immune responses.

## Trained Immunity

Recent advances in innate immune studies show that both myeloid cells such as monocytes, macrophages and dendritic cells display changes in their metabolic and epigenetic programming to become hyperresponsive to second stimulation by the same antigen (8). This *de facto* “innate immune memory” is commonly referred to as “trained immunity” (8). The review by Zhang et al. discusses the role of IMMs such as β-glucans and TLR ligands in trained innate immunity in fish. Additionally, they describe how trained immunity can be introduced in brood stock fish and their offspring, highlighting its potential application as an alternative method to conventional vaccination.

## Antigen Sampling Cells

Antigen uptake through mucosal surfaces by specific antigen sampling cells (ASCs) is well-documented in mammals where it is mainly done by M-cells (9). However, specific ASCs have not been described in fish despite the identification of several mucosal tissues that include the gills, skin, the digestive tract and the nose. To gain further insight on antigen uptake at mucosal surfaces, a key process for the optimization of mucosal vaccines, Kato et al. identified two phenotypes of ASCs able to take up antigens through rainbow trout gills. One phenotype had large vacuoles in the cytoplasm and expressed CD45, MHC-II, CD83, IL-1β, and IL-12p40b. Morphologically, this subset features of monocyte, macrophage and dendritic cells. The second phenotype exhibited features similar to mammalian M-cells that bind lectin UEA-1 but not WGA and expressed the surface marker Anxa5. Unlike mammalian M-cells, teleost M-like-cells possessed small vacuoles in the cytoplasm, but played a role in antigen sampling using mechanisms similar to the bona-fide M-cells and expressed genes linked to phagosome, lysosome, and antigen processing and presentation pathways seen in mammalian M-cells.

## Identification of Protective Antigens for Vaccine Design

Pathogen diversity poses a challenge for the selection of vaccine candidates with broad protective ability across variant strains. To this aim, various techniques aimed at identifying vaccine antigen candidates with broad protective abilities are being sought. For example, Wang Y. et al. used the isobaric tag for relative and absolute quantification (iTRAQ), to identify protective biomarkers among 341 *Aeromonas hydrophila* genes expressed in response to iron starvation. By knocking out three genes that had low protease activity, they constructed three avirulent mutants as live vaccines that produced high protection in zebrafish (*Danio rerio*). Another approach used to identify broadly protective antigens is by performing multiple sequence alignments to identify conserved antigenic proteins protective across variant strains. Wang E. et al. used this approach to design a *Yersinia ruckeri* outer membrane porin F (OmpF) vaccine after identifying a highly conserved motif among 15 sequences of *Yersinia* species, which confered increased protection and high antibody responses in channel catfish. Similarly, the paper by Maiti et al. provides a detailed account of various *in silico* and bioinformatics analytical tools used to identify outer membrane protein (OMP) antigens with broad protective abilities against variant strains of different bacterial species infecting major farmed fish species in India.

## Live Attenuated and Recombinant Vaccines

Although there are many commercial antibacterial vaccines available for use in aquaculture, there are still some bacterial pathogens for which vaccines have not been developed or are deficient. In this Special Issue, different recombinant approaches have been explored. Abdelhamed et al. produced a recombinant *A. hydrophila* ATPase protein vaccine that showed increased survival and reduced bacterial concentrations in different tissues of catfish after challenge with virulent *A. hydrophila* ML09-119 strain. Lange et al. showed a progressive increase of systemic and mucosal IgM levels over a period of 2 years in catfish immunized by bath using a recombinant DnaK (rDnaK) vaccine made from the *Flavobacterium columnare* DnaK heat-shock-protein (Hsp70 protein). Finally, Maiti et al. reviewed the use of outer membrane proteins (OMPs) as subunit, DNA and nanoparticle vaccines against different pathogens infecting farmed fish in India. In the case of intracellular replicating bacteria, an efficient vaccine should be able to evoke both humoral and cell-mediated immune responses able to eliminate infected cells. To this end, Kordon et al. developed two live attenuated vaccines (LAVs) designated as *Ei*Δ*evpB*, and ESC-NDKL1 against *Edwardsiella ictaluri*, a facultative intracellular replicating bacterium. Both LAVs elevated IFN-γ expression in lymphoid organs that correlated with increased CD207^+^ cells and polarization of T-helper genes in vaccinated catfish.

## Temperature Manipulation and Host Metabolism

Temperature manipulation is an important factor known to alter virulence of various fish pathogens (10) as well as the immune responses of the fish host (11). Although several studies have shown that virulence factors can be controlled by thermo-sensors in different bacterial pathogens (10, 12), little is known regarding how modulating temperature changes influence the ability of the host responses to mount protective immune responses against invading pathogens. The study undertaken by Jiang et al. showed that crucian carp (*Carassius carassiuss*) cultured at 30°C showed an increased tricarboxylic acid (TCA) cycle linked to reduced taurine and hypotaurine metabolism coupled with low unsaturated fatty acid biosynthesis, which enhanced bacterial infection in a dose-dependent manner in fish. Conversely, exogenous treatment of fish using these metabolites increased fish survival and rescued the decline of pro-inflammatory cytokine expression that included TNF-α1, TNF-α2, IL-1β1, IL-1β2, and lyz expression. This study shows that changes in the interplay between temperature and fish metabolism can be used to regulate immune protection against bacterial infection in fish.

## B Cell Immune Responses in Vaccinated Fish

Antibody production together with cell mediated immune responses constitute the hallmark of long-term protective immunity in vaccinated fish. As such, antibodies produced by B cells, are widely used as a measure of immune response to vaccination in fish (4). Along this line, Teige et al. developed a multiplexed bead antibody assay able to differentiate antibody responses induced by piscine reovirus (PRV) σ1, σ2, and σ3 antigens. In trying to elucidate factors driving the specificity of fish antibodies, Magadan et al. searched for “public” antibody clonotypes common to all individuals that may mediate universal protection against pathogens. Through CD3 spectratyping of splenic B cells 5 months after vaccination with an attenuated viral haemorrhagic septicaemia virus (VHSV) vaccine, they showed that the IgM repertoires of vaccinated fish had altered profiles for the heavy chain variables (VH) VH1, VH4, and VH5 families. The altered profile of VH5 was the only clonotype found in all vaccinated individuals, suggesting that it may contain a persisting public component, while responses associated with VH1 and VH4 varied from fish to fish. Further analysis of the variable (V), joining (J), and diversity (D) gene segments showed that the VH5 and JH5 clonotypes formed persistent public VH5JH5 IgM dominant antibodies common to all vaccinated individuals. In addition to these studies, Peñaranda et al. obtained a cell surface proteome of Atlantic salmon IgM^+^ B cells by mass spectrometry and comparing it to that of two adherent salmon head kidney cell lines and used it to identify specific markers of B cells, namely CD22 and CD79A, in salmon. The identification of these molecules is very important to track B cell responses to vaccines and/or immunostimulants in the future.

## Measures of Protective Immunity for Fish Vaccines

To a large extent, the correlates of protective immunity for most fish vaccines are unknown. Moreover, whether common signatures of protective immunity across different fish species can be identified or not remains a challenge. However, novel high throughput sequencing (HTS) techniques such as RNA-seq that provide whole transcriptomes of immune genes expressed in response to different stimuli are beginning to unravel different molecular markers that could serve as signatures of protective immunity expressed by different fish species in response to different pathogens. The study performed by Maekawa et al. describes the use of RNA-seq as a useful tool used to identify the universality and diversity of immune responses against different pathogens expressed in different fish species by comparing immune genes expressed in response to *Nocardia seriolae* in largemouth bass (*Micropterus salmoides*), *Lactococcus garvieae* in gray mullet (*Mugil cephalus*), *Vibrio harveyi* in orange-spotted grouper (*Epinephelus coioides*), and *Aeromonas sobria* in koi carp (*Cyprinus carpio*). They found 39 differentially expressed immune genes that were present in all fish species having the potential to serve as universal immunological markers of protective immunity. This study suggests that common signatures of protective immunity can be identified for use across different fish species.

Overall, the ccontributions gathered in this Special Issue provide a wide range of novel approaches and technologies being developed aimed at producing highly protective vaccines for finfish. What is now required is translation of these experimental findings to the fish farming industry with the objective of reducing the disease burden in aquaculture through vaccination.

## Author Contributions

All authors listed have made a substantial, direct and intellectual contribution to the work, and approved it for publication.

## Conflict of Interest

The authors declare that the research was conducted in the absence of any commercial or financial relationships that could be construed as a potential conflict of interest.
